# Recent Strategies to Attenuate Hepatocellular Carcinoma Recurrence After Liver Transplantation: A Narrative Review

**DOI:** 10.3390/cancers17101650

**Published:** 2025-05-13

**Authors:** Yutaka Endo, Yuki Bekki, Roberto Hernandez-Alejandro, Koji Tomiyama

**Affiliations:** Department of Transplant Surgery, University of Rochester Medical Center, Rochester, NY 14626, USA; yutaka_endo@urmc.rochester.edu (Y.E.); yuki_bekki@urmc.rochester.edu (Y.B.); roberto_hernandez@urmc.rochester.edu (R.H.-A.)

**Keywords:** hepatocellular carcinoma, liver transplant, recurrence

## Abstract

Hepatocellular carcinoma (HCC) is one of the leading causes of liver transplant worldwide. While liver transplantation offers a survival advantage for early-stage HCC patients, post-transplant recurrence remains a significant concern, affecting up to 15% of recipients. We sought to conduct a comprehensive review related to HCC recurrence after liver transplant. Tumor-related factors such as poor differentiation, vascular invasion, and elevated tumor biomarkers like alpha-fetoprotein are key predictors of recurrence. Donor-related factors, including graft type and surgical procedures, can also influence outcomes, though their effects are less conclusive. Advancements in patient selection criteria and scoring systems, such as the Milan Criteria and RETREAT score, have improved risk stratification by incorporating tumor size, biomarkers, and response to pre-transplant treatment. Despite these measures, recurrent HCC after transplantation poses treatment challenges. Curative approaches such as resection are feasible for localized or oligometastatic recurrence and offer the best outcomes when applicable. Locoregional treatments, including ablation and transarterial chemoembolization, provide options for unresectable cases but have limited long-term efficacy. Systemic therapies, including targeted agents like sorafenib, regorafenib, and lenvatinib, have shown modest benefits in managing advanced recurrent HCC. Emerging immunotherapy approaches hold promise but face unique challenges due to the required immunosuppression in transplant recipients. Multidisciplinary evaluation remains essential for tailoring treatment plans. Future efforts should focus on refining predictive tools and exploring novel therapies to improve survival outcomes for patients with recurrent HCC after liver transplantation.

## Simple Summary

Hepatocellular carcinoma (HCC) is one of the main reasons for liver transplants, but up to 15% of patients experience recurrence after surgery. Factors like poor tumor differentiation, vascular invasion, and high alpha-fetoprotein levels increase the recurrence risk. Donor-related factors also play a role, though their impact is less clear. Selection criteria, such as the Milan Criteria and RETREAT score, help predict recurrence risk. Treatment options for recurrent HCC include surgery for localized cases, locoregional therapies like ablation and chemoembolization, and systemic treatments such as targeted drugs (sorafenib, regorafenib, lenvatinib). Immunotherapy shows potential but is complicated by the need for immunosuppression. A multidisciplinary approach is key to managing recurrence, and future research should focus on better prediction tools and new therapies to improve patient outcomes.

## 1. Introduction

Hepatocellular carcinoma (HCC) is the sixth most prevalent malignancy globally, with a poor 5-year survival rate of 21% [1]. The recent surge in HCC incidence is becoming a major concern for healthcare providers in the United States [1,2]. The Barcelona Clinic Liver Cancer (BCLC) guideline recommends considering liver transplantation as the first option for early-stage hepatocellular carcinoma (BCLC-A) if the patient is eligible [3]. This recommendation is reflected by past studies that have shown liver transplantation offers a survival benefit compared to other treatment modalities [4,5]. HCC has emerged as a primary driver for liver transplantation (LT), with a steady rise in transplant demand. In recent years, HCC has accounted for approximately 15% of LT indications in the USA [6,7]. Given the characteristics of a liver transplant, where a donor is inevitably involved, selecting optimal candidates for liver transplant is crucial [8]. Due to the circumstances, patient selection criteria, starting with Milan Criteria, have been developed and utilized to select optimal candidates, and the scoring system has been a target of investigation to balance between widening the eligibility and minimizing the risk of recurrent HCC [9,10]. Nonetheless, despite these efforts, HCC recurrence remains a substantial problem, with recurrence rates approximately 15% with a median time to recurrence of just over 1 year [11]. Since LT removes a cirrhotic liver where HCC may recur de novo, post-transplant tumor recurrence is thought to result from undetected extrahepatic metastases present before transplantation or from the release of circulating tumor cells [12,13]. Therefore, selecting an appropriate candidate as well as donor and performing a safe procedure are both crucial. In addition, survival after recurrence is usually deemed unsatisfactory due to limited treatment options as well as a necessary immunosuppressed state and is typically shorter than survival after recurrence after surgical liver resection [11,14,15,16]. Therefore, better patient selection criteria, detecting risk factors for worse outcomes, and exploring effective post-recurrence treatment strategies would be associated with optimizing the post-transplant survival benefit [17]. In summary, this review will focus on up-to-date risk factors and risk scoring systems for post-LT HCC recurrence, as well as the treatment modalities for recurrent HCC.

## 2. Risk Factors for Post-LT HCC Recurrence

### 2.1. Recipient and Tumor-Related Factors

Most investigated determinants of post-transplant survival have been tumor-related factors [18]. In an early report, the tumor differentiation at the explant had a prognostic impact among HCC-LT patients [19]. This study showed that LT recipients with poorly differentiated HCC had a worse survival outcome compared to individuals with well-differentiated or moderately differentiated HCC. An analysis conducted on data from the Organ Procurement and Transplantation Network/United Network for Organ Sharing (OPTN/UNOS) registry, covering the period from April 2012 to December 2014, highlighted several significant pre-transplant factors regarding explantation that strongly predict recurrence risk. These factors include the presence of extrahepatic spread or lymph node involvement, poor tumor differentiation, and both microvascular and macrovascular invasion. Additionally, tumors classified under explant TNM stages T4 or T3, downstaging from a stage higher than T2, and elevated alpha-fetoprotein (AFP) levels were also identified as strong predictors [20]. A similar finding that explant pathological data are a crucial determinant has been observed in Italy. Lasagni et al. compared those with recurrent HCC with a matched pair of HCC patients without recurrence, investigating the impact of expression of angiopoietin-2 [21]. Individuals who experienced HCC recurrence exhibited significantly elevated expression of angiopoietin-2 in the tumor endothelium upon pathological examination. Multivariable analysis confirmed that this gene’s expression was an independent determinant of recurrence (HR: 5.63, 95% CI: 2.60–12.3, *p* < 0.001) [21]. This underscores the role of tumor-related factors, which have been strongly associated with HCC recurrence post-transplant, in explant pathology.

On top of the explant pathology, a serum tumor biomarker related to HCC recurrence has been investigated as it may have the potential to help surgeons to stratify patients preoperatively. Prior tumor markers such as AFP at the time of LT are known to be a potent predictor of post-transplant recurrence [22,23,24]. Recent studies demonstrated that Des-gamma-carboxyprothrombin (DCP) and AFP bound to Lens culinaris agglutinin (AFP-L3) were more accurate biomarkers for predicting post-transplant HCC recurrence. In a prospective study involving 285 recipients meeting the Milan Criteria, AFP-L3 and DCP emerged as the most powerful determinants of recurrence after transplant. Notably, when both tumor markers surpassed their criteria (AFP-L3 ≥ 15% and DCP > 7.5), the markers accounted for 61.1% of all recurrence cases, underscoring their predictive power [25]. Interestingly, risk scoring systems incorporating these biomarkers demonstrated relatively better predictive values (c-index 0.75~0.97) than existing risk models (overall less than 0.7) [26,27]. In recent years, liquid biopsy using cell-free DNA has shown potential to serve as a more precise biomarker for detecting recurrence after liver transplant among HCC recipients [28].

An inflammation marker has been found to be one of the determinants of tumor recurrence, as HCC may induce an inflammatory response that increases the release of cytokines and inflammatory mediators [29]. A meta-analysis suggested that a high neutrophil-to-lymphocyte ratio (NLR) was a surrogate marker for microvascular invasion, multifocal tumor, larger tumor size, poor differentiation, and reduced overall survival [30]. Recipient age is also a key determinant of survival, especially for patients older than 65 years, according to a previous report [31]. Additionally, the presence of underlying liver disease and other comorbidities has been shown to strongly influence prognosis. In HCC patients with hepatitis B-related liver disease, coexisting diabetes mellitus is associated with a higher risk of tumor recurrence [32]. Similarly, recipient obesity has been linked to an increased recurrence risk [33,34]. In contrast, metabolic-dysfunction-associated steatotic liver disease has been associated with a more indolent tumor biology and a lower likelihood of recurrence following liver transplantation [35,36]. Importantly, previous studies have demonstrated that treating the underlying liver disease—particularly hepatitis C virus (HCV) infection—can significantly reduce the risk of recurrence [37,38]. Therefore, managing the recipient’s background liver disease is crucial in minimizing post-transplant tumor recurrence. These factors mentioned above are summarized in Table 1.

### 2.2. Donor-Related or Surgery-Related Factors

Several donor-related factors have been reported as determinants of a higher incidence of recurrence [39]. Donor age has been a well-investigated topic in this field; a prior study demonstrated a higher median age of the donor among patients who evolved with recurrence after LT among HCC patients during 2004–2011 [39]. The explanation of this would be derived from that older livers would be vulnerable to cold ischemia and ischemic reperfusion injury (IRI), which may be correlated with susceptibility to tumor recurrence [40]. The mechanism behind this could be derived from that an IRI may stimulate immune and inflammatory reactions, which would impact tumor recurrence [41]. This is in line with another study’s finding that incrementally prolonged ischemia time was associated with a gradual increase in tumor recurrence risk [42]. On the other hand, a study utilizing a more recent cohort revealed that older donor age was associated with decreased OS but not liver-specific survival as well as post-transplant tumor recurrence in HCC recipients. Donor age also had different effects in patients with different underlying liver diseases [43]. These conflicting results would suggest that a more complex mechanism could be underlying the association between donor age and post-transplant HCC recurrence. Another possible determinant of outcome is donor sex. Of note, the lowest rates of HCC recurrence were confirmed among the group of female recipients of male donor grafts in the deceased donor LT cohort [44]. While the mechanism behind it has been still unclear and the donor’s sex likely does not have a significant impact on donor selection, this will require further consideration in future studies.

The choice of a liver graft remains a topic of debate. With the rapid advancements in machine perfusion techniques, donation after circulatory death (DCD) has emerged as a viable option, particularly in regions facing organ shortages [45]. To date, multiple studies from various regions consistently demonstrate that DCD-LT offers similar recurrence-free survival (RFS) when compared to donation after brain death liver transplantation (DBD-LT) [46,47,48,49]. A recent meta-analysis further confirmed that comparable outcomes can be achieved in HCC patients undergoing DCD-LT versus DBD-LT [50]. Interestingly, the data from two institutions suggested that the use of DCD donors was associated with poorer OS but comparable RFS [51]. Therefore, the decision between DBD and DCD may have minimal impact on oncological outcomes but remains closely tied to patient survival. Recently, the use of machine perfusion for DCD donors has increased; however, its effect on HCC recurrence remains unclear. Another graft option could be a living donor (LD). Of note, there have been numerous conflicting results regarding outcomes between LDs and cadaveric donors [52,53,54,55,56,57,58,59]. Some earlier studies have suggested that LDLT may be associated with a higher recurrence rate compared to DDLT. However, this discrepancy is likely due to the inability to assess tumor biology during the waitlist period in patients receiving LDLT, unlike those receiving DDLT [58,59]. For instance, Park et al. reported a higher risk of recurrence in LDLT than in DDLT among high-risk patients [58]. Conversely, Vakil et al. found that survival following LDLT was significantly better than survival following DDLT for HCC during the same period, although their cohort size was small [55]. Furthermore, a meta-analysis revealed no significant differences in disease-free survival (DFS) and recurrence rates after transplantation between LDLT and DDLT [60]. Rather, prior studies supported LDLT in terms of intention-to-treat analysis as the dropout rate for potential LDLT recipients was lower than that for DDLT patients [61]. To that end, LDLT may contribute to reducing the dropout rate and subsequently improve the outcome in intention-to-treat analysis compared to DDLT, but they are likely to be comparable from an oncologic standpoint.

Another important surgical consideration is the hepatic vein reconstruction technique. There are two primary approaches: piggyback and bicaval reconstruction [62]. In general, preserving the vena cava in piggyback procedures reduces hemodynamic instability and minimizes warm ischemia time [62]. On the other hand, preserving the cava theoretically increases the risk of tumor-affected margins persisting, and the extensive manipulation of the patient’s liver may promote the spread of tumor cells [63]. Research on this topic has produced mixed results. Mangus et al. found no significant difference in recurrence rates or RFS between the two reconstruction techniques [64]. Conversely, a Polish study reported a higher risk of recurrence in patients undergoing the piggyback maneuver [65]. More recent multicenter analyses from Europe support the use of the total caval replacement technique. These studies indicate that conventional piggyback reconstruction is significantly associated with a higher incidence of tumor recurrence compared to bicaval anastomosis [63]. It is worth noting that these findings are primarily based on European studies, with limited multicenter data from other regions, including North and South America, as well as Asia. Consequently, further research is needed to generalize surgical recommendations for hepatocellular carcinoma and liver transplantation globally.

### 2.3. Patient Selection and Risk Scoring Systems

Given the profound effect of HCC recurrence on post-transplant outcomes, scoring systems for patient selection have been largely shaped by factors assessed both before and after LT [66]. Transplant selection models in the early years, like the Milan Criteria, prioritized only tumor size and lesion count [67]. However, newer frameworks—such as the UCSF and up-to-7 criteria—have demonstrated that successful liver transplantation is possible even when extending beyond Milan’s rigid boundaries [68,69]. Aside from tumor morphology, another important factor to consider would be tumor biology, and AFP has been generally used as a surrogate. Of note, several scoring systems that were reported to differentiate post-transplant survival successfully have incorporated AFP into tumor size/numbers [8,23,70,71]. In other studies, Halazun et al. demonstrated that a score using tumor size, AFP, and neutrophil-to-lymphocyte ratio successfully classified recipients according to the recurrence risk (four groups; low to very high), while Sasaki et al. integrated the tumor burden score and MELD-Na score as well as AFP [9,72]. In the era of neoadjuvant treatment, one report from the USA demonstrated that the response in AFP level would increase the predictive value regarding post-transplant HCC recurrence [73].

A concise and comprehensive risk stratification system, namely the Risk Estimation of Tumor Recurrence After Transplant (RETREAT) score, was designed to assess the likelihood of HCC recurrence in patients who have undergone transplantation for HCC [10]. This score incorporates the presence of microvascular invasion, the sum of the largest diameter of viable tumors, the number of viable tumors, and AFP levels at transplant. The RETREAT score ranges from 0 to 8, stratifying recurrence risk from very low (score 0, 5-year recurrence risk of 2.9%) to extremely high (score ≥ 5, 5-year recurrence risk of 75.2%). A surveillance schedule used by numerous institutions, including University of Rochester Medical Center, suggests the following: no surveillance for patients with a RETREAT score of 0 (given the low 3% 5-year recurrence rate), surveillance every 6 months for 2 years for scores of 1–3, every 6 months for 5 years for a score of 4, and intensified surveillance (every 3–4 months for 2 years, then every 6 months for years 2–5) for scores ≥ 5 [17]. Similarly, our institution utilized the RETREAT score as a guide for surveillance.

## 3. Treatment Options for HCC Recurrence Post-LT

Treatment strategies after HCC recurrence in the post-LT setting have not been well studied, and a comprehensive consensus has been limited. Considering the treatment pattern of recurrent HCC after liver resection, treatment options depend on the recurrent site and numbers/size [15,74]. Also, in general, patients with intrahepatic recurrence or recurrence in other “resectable” recurrent sites (i.e., lung, adrenal) in a solitary or oligometastatic fashion are likely to have curative-intent re-resection, while those with unresectable recurrent diseases tend to receive other locoregional treatments or systemic treatments. One important point is that while de novo recurrence can occur after liver resection, it does not happen after liver transplantation. Recurrence after liver transplantation is basically related to systemic circulation. Therefore, compared to recurrence after liver resection, the treatment options may be relatively limited.

### 3.1. Resection

Resection for recurrent HCC lesions after LT has been reported to offer the best survival benefits among various treatment options, provided it is feasible. Previous reports indicated that surgery for recurrent lesions achieved the best long-term survival rates compared to other treatment modalities [14]. Notably, the 3-year survival rate of surgery was much better than that of nonsurgical therapy, including ablative/interventional radiology therapy (surgery: 60% vs. nonsurgical modalities: 11%). Another retrospective study by Sapisochin et al. revealed that found that patients with recurrent HCC after LT who underwent curative treatments, such as resection or ablation, had significantly higher survival rates compared to those who received palliative therapies, including systemic chemotherapy, sorafenib, or best supportive care [75]. The most updated study from North America highlighted the success of hepatic resection in managing recurrent HCC following liver transplantation [76]. Over a median follow-up of 50.7 months, patients who underwent resection for recurrent HCC achieved a median overall survival of 49.6 months, indicating promising long-term outcomes. However, these studies likely exhibit some selection bias, as the best outcomes were typically seen in patients with unifocal, often extrahepatic, disease that was more amenable to surgical resection.

Although the number of patients is too small to draw solid conclusions, several studies have demonstrated that extrahepatic metastatectomy could be applicable in an oligometastatic setting (Table 2) [77,78,79,80,81]. For limited pulmonary metastases, the excision of lung lesions was associated with favorable outcomes. Results from the largest single-center cohort (*n* = 52) in South Korea showed relatively favorable OS (1-year OS: 75.0%, 3-year OS: 43.5%, 5-year OS: 33.9%) after pulmonary resection. This study also found that the timing of recurrence, elevated AFP at metastatectomy, and adjuvant chemotherapy after lung resection were correlated with worse OS [81]. In addition, there have been reports of successful extrahepatic metastatectomy; however, these reports were limited to case series and case reports, with low-level evidence [82,83,84]. Therefore, while the presence of extrahepatic recurrence does not necessarily preclude curative resection, it is more important to carefully evaluate the validity of such cases on a case-by-case basis in multidisciplinary conferences [85].

### 3.2. Locoregional Treatment

Ablation treatment is considered a potential option for patients with unresectable recurrence following LT. However, the body of research on this approach is limited, particularly in terms of the size of study populations. Zhai et al. demonstrated that microwave ablation has been identified as a safe technique for treating post-LT recurrence, assessing 11 patients, and reported no significant complications [86]. In another retrospective study, Huang et al. found comparable 5-year OS (surgery: 35%, vs. RFA: 28%, *p* = 0.88) but worse 5-year DFS with RFA (surgery: 16%, vs. RFA: 0%, *p* = 0.75), although these results were not significant [87]. The evidence regarding the efficacy of ablation therapy for recurrent HCC remains under-researched and has yet to be thoroughly evaluated in larger studies or compared with other treatment modalities.

Regarding transarterial chemoembolization (TACE), Ko et al. reported on the outcomes of recurrent HCC following living donor LT treated with a cisplatin infusion followed by a mixture of iodized oil and cisplatin [88]. The study reported a complete or partial response to TACE in 50% of the patients. However, a significant proportion of patients (93%) experienced the recurrence of HCC within six months, and around 20% had disease progression despite undergoing TACE. The median survival following TACE was reported to be 9 months. Similar results were reported by Zhou et al., showing improved post-recurrence survival compared to those who did not receive such treatment (9 months vs. 3 months, respectively) [89].

### 3.3. Systemic Treatment

Management of post-LT recurrence remains a challenging area due to limited research on the efficacy of various cytotoxic chemotherapeutic regimens, as shown in Table 3. Sorafenib, known for its efficacy in managing advanced primary HCC, has been investigated for treating non-resectable recurrent HCC post-LT [90,91,92,93,94]. A meta-analysis by Mancuso et al., which reviewed eight studies, reported a median survival of 10.5 months (range: 5–21.3 months) and a pooled 1-year survival rate of 63% (range: 18–90%) [95]. However, sorafenib use in the post-transplant setting is frequently limited by side effects, with up to 50% of patients requiring dose reductions in some studies [96]. Regorafenib, another multikinase of choice for unresectable HCC, has been considered as a second-line drug for patients with disease progression while on sorafenib [97]. A multicenter retrospective study involving 28 LT patients with recurrent HCC who progressed on sorafenib found regorafenib to be well tolerated. While all patients experienced adverse events, the most common grade 3/4 events were generally manageable, encompassing fatigue and dermatological reactions. Lenvatinib, another oral multikinase inhibitor, may be effective for post-LT HCC recurrence; a multicenter retrospective study demonstrated that its efficacy and toxicity profiles in these patients were consistent with those observed in non-LT HCC patients in prior studies [98,99].

### 3.4. Immunotherapy

Checkpoint inhibitors for immunotherapy represent a promising avenue for advanced primary HCC [101,102,103]. In the context of recurrent HCC post-LT, treatment with checkpoint inhibitors requires caution due to an elevated risk of allograft rejection [104]. This was confirmed by the study conducted at the Mayo Clinic that investigated the PD-1 inhibition effect among patients with a history of LT [100]. In this study, three of seven patients discontinued therapy early due to graft rejection or multi-organ failure. Literature reviews report graft rejection rates ranging from 25 to 54%, with rejection typically occurring rapidly (8–19 days after initiation of immunotherapy) [105]. A case report describes how a recipient who received an ICI shortly before LT experienced severe liver necrosis [106]. Nonetheless, the small number of patients and variability in studies may prevent us from reaching a consensus on the optimal immunosuppression regimen for preventing graft rejection or the best treatment for rejection if it occurs. Personalized assessment of allograft PD-1 lymphocyte expression may enable better patient selection, optimizing the risk–benefit ratio for checkpoint inhibitor therapy [107]. Another possible solution is a dedicated immunotherapy and immunosuppression plan. One large multicenter study suggested that rejection was predominantly seen in patients whose last dose of ICI was within 3 months of LT [108]. This may indicate that an adequate washout period would be needed not to compromise the risk of rejection, yet the optimal period has not been elucidated. On top of that, adjustment of immunosuppression might help to prevent severe rejection after ICI [109]. But again, this is ill defined as of now.

## 4. Conclusions

In conclusion, HCC recurrence following LT remains a significant challenge despite advancements in patient selection criteria, risk scoring systems, and treatment strategies. Tumor-related factors, donor characteristics, surgical techniques, and biomarker profiles play critical roles in predicting recurrence risk and tailoring post-transplant management. Emerging evidence highlights the importance of personalized approaches, such as integrating tumor biology and biomarkers like AFP and DCP into scoring systems, to improve prognostic accuracy. While surgical resection offers the best outcomes for recurrent HCC when feasible, systemic therapies continue to evolve as viable alternatives for managing unresectable cases. Ongoing research into optimizing candidate selection, refining recurrence risk assessment, and expanding treatment options is essential to enhance overall survival and recurrence-free survival for HCC patients undergoing LT.

## Figures and Tables

**Table 1:** Recipient and donor/surgical factors determining the oncological outcome after liver transplant.

**Determinants of Outcome**
*Recipient factor*
HCC number and size
Vascular invasion
Degree of differentiation
Tumor biomarkers
Neutrophil–lymphocyte ratio
Patient age
Obesity
Hepatitis C treatment
Diabetes Mellitus
Metabolic-dysfunction-associated steatotic liver disease
*Donor- and procedure-related factors*
Donor age
Donor sex
Ischemic time
Surgical technique
Types of graft

**Table 2:** Selection criteria for hepatocellular carcinoma liver transplant.

**Selection Criteria**	**Year**	**Country**	**Tumor Morphology**	**Serum Marker**	**Additional Features**	**Outcomes**
Milan Criteria	1996	Italy	Single tumor > 5 cm or ≤3 tumors ≤ 3 cm	-	No vascular invasion or lymph nodes	Within Milan Criteria 5-year OS: 85% 5-year RFS: 92%
UCSF Criteria	2001	USA	Single nodule ≤ 6.5 cm or 2–3 nodules ≤ 4.5 cm and total diameter ≤ 8 cm	-	No vascular invasion or lymph nodes	5-year OS: 75.2% 5-year RFS 80.9%
Up-to-7 Criteria	2009		Sum of the largest tumor size and number of lesions < 7		-	5-year OS: 71.2% (beyond Milan and within up-to-7 criteria)
French AFP	2012	France	Size: 1: ≤3 cm, 2: 3–6 cm, 3: >6 cm Number: 1: 1–3, 2: ≥4	AFP: 1: ≤100, 2: 100–1000, 3: >1000 ng/mL	-	Low-risk (score ≤ 2): 5-year RFS: 86.6% 5-year OS: 69.9% High-risk (score > 2): 5-year RFS: 54.7% 5-year OS: 40.8%
Metroticket 2.0	2018	Italy?	HCC within up-to-7 criteria if AFP < 200 ng/mL; HCC within up-to-5 criteria if AFP 200–400 ng/mL; HCC within up-to-4 criteria if AFP 400–1000 ng/mL		-	Within criteria 5-year RFS: 89.6% 5-year OS: 79.7% Beyond criteria 5-year RFS: 46.8% 5-year OS: 51.2%
Pre-MORAL	2017	USA	Largest tumor size > 3 cm	Maximum AFP > 200 ng/mL Preoperative NLR ≥ 5	-	5-year RFS Low-risk: 98.6% Medium-risk: 69.8% High-risk: 55.8% Very high-risk: 0%
RETREAT	2017	USA and Canada	Sum of the largest tumor size and number (0, 1.1–4.9, 5–9.9, 10<)	AFP (0–20, 21–99, 100–999, >1000 ng/mL)	Microvascular invasion	5-year recurrence risk Score 0: 2.9% Score 5: 75.2%
HALT-HCC	2017	USA	Tumor burden score	Natural logarithm of AFP	MELD-Na	Risk equation: 1.27 × TBS + 1.85 × ln(AFP) + 0.26 × MELD-Na 5-year OS: Q1: 78.7% vs. Q2: 74.5% vs. Q3: 71.8% vs. Q4: 61.5%
NYCA criteria	2018	USA	Maximum tumor size Maximum tumor number	AFP response (max to final)	-	5-year RFS: Low-risk (score 0–2): 90% Acceptable-risk (score 3–6): 70% High-risk (score ≥ 7): 42%
Japanese Criteria “5-5-500”	2019	Japan	Tumor size (≤5 cm) and tumor number (up to 5)	AFP ≤ 500 ng/mL	-	Within criteria 5-year RFS: 73.2% 5-year OS: 75.8% Beyond criteria 5-year RFS: 43.4% 5-year OS: 52.1%
Seoul Criteria	2007	South Korea	Tumor size (≤3, 3.1–5, 5.1–6.5, >6.5 cm) and number (1, 2–3, 4–5, >5)	AFP (≤20, 20.1–200, 200.1–1000, >1000 ng/mL)	-	Score 3–6 (transplantable): 3-year RFS: 87% 3-year OS: 79% Score 7–12 (non-transplantable): 3-year RFS: 31% 3-year OS: 38%

AFP, alpha-fetoprotein; OS, overall survival; RFS; recurrence-free survival.

**Table 3:** Summary of previous studies related to several treatment strategies for recurrent HCC after liver transplant.

**Publication**	**Year**	**Study Cohort**	**Outcomes**
***Surgical resection***			
Sapisochin et al. [75]	2015	Curative resection; *n* = 38 Palliative treatment; *n* = 51 Best supportive care; *n* = 32	Amenable to curative resection, HR 4.7 (95% CI 2.7–8.3) *
Bodzin et al. [14]	2017	Surgery alone; *n* = 6 Surgery + nonsurgical; *n* = 19 nonsurgical; *n* = 63 Best supportive care; *n* = 18	3-year OS Surgery alone, 60% Nonsurgical treatments alone, 11%
Matar et al. [76]	2024	Hepatectomy for recurrent HCC; *n* = 35	Median RFS, 21.5 months Median OS, 49.6 months
Bates et al. [77]	2008	Pulmonary resection for HCC recurrence; *n* = 5	Median OS, 27.5 months
Han et al. [78]	2010	Pulmonary resection for HCC recurrence; *n* = 41	5-year OS, 66.9% 5-year RS, 24.5%
Hwang et al. [79]	2012	Pulmonary resection for HCC recurrence; *n* = 23	5-year OS, 44.7%
Invenizzi et al. [80]	2020	Pulmonary resection for HCC recurrence; *n* = 4	5-year OS, 43%
Jeong et al. [81]	2021	Pulmonary resection for HCC recurrence; *n* = 52	5-year OS, 33.9%
***Locoregional therapy***			
Zhai et al. [86]	2015	Microwave ablation; *n* = 11	24-months OS, 15.3%
Huang et al. [87]	2015	Surgical resection; *n* = 15 RFA; *n* = 11	5-year OS Surgical resection, 35% RFA, 28%
Ko et al. [88]	2007	TACE; *n* = 28	3-year OS, 6.0%
Zhou et al. [89]	2010	TACE; *n* = 14	24-months OS, 22.2%
***Systemic therapy/Immunotherapy***			
Sotiropoilos et al. [92]	2012	Sorafenib; *n* = 14	Median OS, 25 months
Weinmann et al. [94]	2012	Sorafenib; *n* = 11	Median OS, 20.1 months Median RFS, 4.1 months
Sposito et al. [93]	2013	Sorafenib; *n* = 15	Median Os, 21.3 months
Alsina et al. [90]	2014	Sorafenib; *n* = 9	Median OS, 42 months
de’Angelis et al. [91]	2016	Sorafenib; *n* = 15	1-year OS, 60%
Iavarone et al. [97]	2019	Regorafenib after sorafenib failure; *n* = 28	Median OS from regorafenib, 12.9 months Median OS from sorafenib, 38.4 months
Bang et al. [98]	2023	Lenvatinib; *n* = 45	Median OS, 14.5 months Median PFS, 7.6 months
DeLeon et al. [100]	2018	ICI; *n* = 5	-

* statistical significance.

## Data Availability

Dataset available on request from the authors.
